# Experiences of housing support in everyday life for persons with schizophrenia and the role of the media from a societal perspective

**DOI:** 10.3402/qhw.v11.30571

**Published:** 2016-05-09

**Authors:** Henrika Jormfeldt, Malin Hallén

**Affiliations:** School of Health and Welfare, University of Halmstad, Halmstad, Sweden

**Keywords:** Housing support, media, narratives, persons diagnosed with schizophrenia, social sciences

## Abstract

**Background:**

The mental health-care system in Sweden, as in many other counties, has its main focus on the reduction of psychiatric symptoms and the prevention of relapses. People diagnosed with schizophrenia often have significant health issues and experience reduced well-being in everyday life. The social imaginary of mental illness as an imbalance of the brain has implications concerning general attitudes in society. The news media are an important source of information on psychiatric disorders and have an important role in cultivating public perceptions and stigma. News media can contribute to the mental illness stigma and place individuals with mental illnesses at risk of not receiving adequate care and support. The aim of this preliminary study was to describe users’ experiences of housing support in everyday life.

**Results:**

The results revealed three themes of housing support, which were needed, but frequently insufficiently fulfilled in the municipality. The three themes were: “Support to Practice Healthy Routines in Daily Life,” “Support to Shape Meaningful Contents in Everyday Life,” and “Support to Meet Needs of Integrity and Respect.”

**Conclusions:**

The findings support previous studies arguing that current health care and housing support fails to meet basic needs and may lead to significant and unnecessary health risks. Further investigation is needed regarding the links between attitudes to mental illness in society and political and financial principles for health care and housing support for persons with schizophrenia. Further research is needed regarding the role of the media in policymaking concerning health promotion interventions for people diagnosed with schizophrenia.

The mental health-care system in Sweden, as in many other counties, has a strong focus on the reduction of psychiatric symptoms and prevention of relapses (Van Wel & Landsheer, 2011) in which positive dimensions of health have not been viewed as suitable for evidence-based practice in health care (Jormfeldt, 2011). Barriers regarding health promotion among persons with severe mental illness have been associated with stigma, as well as professional and organizational obstacles in the health-care services provided (Ehrlich et al., 2014). It has been argued that the contemporary paradigm, which focuses on neurological explanations regarding mental illness in society, fails to take psychological and relational explanations of mental illness into account (Rose & Abi-Rached, 2013; Williams, Katz, & Martin, 2011). The news media are an important source of information on psychiatric disorders and have an important role to play in cultivating public perceptions and stigma (Klin & Lemish, 2008). Previous research on news media content has established that journalistic coverage of mental illness is largely characterized by inaccuracies, exaggerations, and misinformation and might even contribute to mental illness stigma through negative news content. For example, inaccurate stories on recovery may promote the belief that mental illness cannot be rehabilitated effectively (Wahl, 2003). News media can contribute to the mental illness stigma by negative portrayals of individuals with these illnesses; therefore, making them vulnerable to social rejection and discrimination and at risk of not receiving adequate care and support (Klin & Lemish, 2008). People diagnosed with schizophrenia belong to a risk group for developing metabolic syndrome, cardiovascular disease, type 2 diabetes and reduced life expectancy, as well as reduced well-being in everyday life (Heald et al., 2010; Lassenius, Åkerlind, Wiklund-Gustin, Arman, & Söderlund, 2013). The causes of this risk are avoidable as it is related to lifestyle in terms of inactivity, overweight, smoking, and poor diet as well as the side effects of antipsychotic medication (Comptom, Daumit, & Druss, 2006). The general public, as well as policymakers, frequently do not have sufficient knowledge of current daily living conditions and the prerequisites for health among people with severe mental illness. People with mental disorders and their families are acutely aware of the negative images of mental illness in the news (and entertainment) media. However, their perspectives about the prerequisites for health as successful stories of recovery have rarely been included as sources of news items (Stuart, 2006). The aim of this preliminary study was to describe users’ experiences of housing support in everyday life.

## Materials and methods

The sample in the present preliminary study consisted of the members of the local service user association, The Interest Alliance for Schizophrenia and Allied Disorders. The local association embraces approximately 50 members; involving people diagnosed with schizophrenia, their relatives, and others with an interest regarding the particular diagnosis. Forty-two letters with information about the purpose of the study and an invitation to write down short narratives concerning experiences of municipality support in everyday life were sent to all of the members of the local service user association. The members of the association were asked to recall situations concerning municipality support in topics, such as housekeeping, diet, physical activity, equality and integrity, inclusion in society, participation in care planning, health promotion and illness prevention, and participation in meaningful activities or employment, as these topics where frequently highlighted as problem areas during members′ meetings in the local association and thus appeared to be crucial to health promotion from their perspective. Twenty-four letters with narratives regarding experiences of support in everyday life from the municipality came in return during July and August, 2011. Five of the letters with narratives were written by individuals with personal experiences of housing support due to mental disability, 14 letters were written by relatives of persons receiving community support, and five letters were written by members with experiences of housing support among people diagnosed with schizophrenia from a professional perspective.

The data were analyzed using a qualitative content analysis method, inspired by Graneheim and Lundman (2004), and carried out by the first author. The narratives were read several times to become acquainted with the content. Meaning units were identified, condensed, abstracted, and labeled with a code. The codes were compared with each other in order to identify similarities and differences regarding the content of the narratives in relation to the aim of the study. The findings were evaluated by means of discussions among both authors with regard to each step of the analysis.

## Ethics

According to the Swedish Health Care Act (2003: 460), concerning the Ethical Review of Research Involving Humans, an ethical review was not necessary as participants were recruited among members of the local organization and no sensitive personal information was requested. Instead, only information without links to a specific person regarding experiences of the housing support provided by the municipality was asked for. All of the participants gave their informed consent verbally and by writing down and sending their narratives to the authors. Confidentiality was assured in accordance with World Medical Association Declaration of Helsinki (2013).

## Results

The narratives collected from the members of the local service user association revealed three themes regarding experiences of housing support in everyday life support which were needed, but frequently insufficiently fulfilled through municipality provision. The three themes were as follows: “Support to Practice Healthy Routines in Daily Life,” “Support to Shape Meaningful Contents in Everyday Life” and “Support to Meet Needs of Integrity and Respect.”

### Support to Practice Healthy Routines in Daily Life

Relatives’ narratives indicated a lack of support on the subject of routines concerning housekeeping, diet, and physical activity. Narratives showed an absence of support among service users in getting up in the morning and not staying in bed all day as well as a lack of support to maintain hygiene routines, uphold physical activity, prepare regular meals, and purchase nutritive food.

Several descriptions affirmed lack of support regarding dietary interventions despite the evidence of the lifesaving nature of these interventions. Narratives showed concerns because service users often eat large portions and have an insufficient fiber intake in combination with too much fat and sugar due to the side effects of medical treatment.

Several descriptions revealed that obesity, which is a well-known common issue related to antipsychotic medication among people diagnosed with schizophrenia, was seldom viewed as a serious matter among municipality staff, even though these issues require regular health checks by a physician. One member of the user association—a relative of a person diagnosed with schizophrenia—stated that:

The staff from the municipal social services do not sufficiently recognize the needs of the service users who, as a result, receive too little encouragement to engage in activities and recover.

### Support to Shape Meaningful Contents in Everyday Life

Narratives from both the relatives and from a professional perspective revealed that service users seldom received sufficient support in their daily tasks, which was perceived as a drawback in their efforts to become involved in the society. The housing support provided by the municipality in the service users’ accommodations was described as “storage” instead of care by the service users’ relatives. The realities described by the narratives showed that the service users often became too dependent upon their relatives to be able to participate in society′s basic activities, because support, company, and reassurance from staff were absent. Another issue frequently reported was the nonexistence of support or solutions to service users’ difficulties to use public transport. One relative expressed the following:

The service user does not have the same opportunities to develop their capabilities … they don't get recognized and encouraged enough to carry out things properly. They simply don't get help ….

### Support to Meet Needs of Integrity and Respect

Some narratives spoke of the experiences of understanding and respectful behavior among staff, even though an “us and them” perspective was evident in most staff–user relationships, as well as experiences of inequalities regarding power and decision-making. Narratives of relatives expressed their experiences of violations of both service users’ and their relatives’ dignity when staff were intruding their individual integrity by claiming transparency regarding the service user's health status and personal relationships. Narratives frequently described insufficient knowledge among staff concerning the special difficulties related to the diagnosis such as lack of motivation and side effects of medical treatment. Moreover, they easily accepted service users’ wishes to draw back and isolate themselves without considering the hostile effects of isolation, and the fact that the diagnosis of schizophrenia often involves a tendency to immure oneself. One service user described his experiences as this:

The first years when I became ill I was just assigned to an apartment and left all by myself with prescribed medication. It took me several years of misery before staff paid any attention to my situation and gave me a hand to get me back on track. Now I have got sufficient support from staff but I should have got it from the beginning.

## Discussion

The lack of support regarding positive dimensions of health among people with severe mental illness has been revealed previously (Ehrlich et al., 2014; Jormfeldt, 2011) and, according to the findings of this preliminary study, this topic still needs attention. The results of this preliminary study show that current available housing support for people diagnosed with schizophrenia fails to meet basic needs and may lead to significant and unnecessary health risks and reduced well-being in line with previous studies conducted by Comptom et al. (2006), Heald et al. (2010), and Lassenius et al. (2013). There is a reason to believe that stigma and barriers regarding health promotion among people with severe mental illness (Ehrlich et al., 2014) are related to the negative media portrayals of mental illness (Wahl, 2003). Furthermore, the current focus on the reduction of psychiatric symptoms and prevention of relapses in mental health care could be transformed by media information concerning the fact that mental illness can be rehabilitated effectively (Van Wel & Landsheer, 2011). Further studies are needed regarding the public discourse of mental illness in public debate and within the scientific community to increase awareness of how the current discourse shapes the existing delivery of education, research, and clinical praxis in mental health services. An important topic in future research should be the role of media portrayals of mental illness as it directly affects people with mental illnesses by impeding their social participation and interferes with their recovery (Klin & Lemish, 2008). In addition, further research is required regarding the perspective of professionals in mental health services and journalists focusing on mental health and illness.
